# An exploratory study of the level of folic acid in food staples in Ireland in 2021

**DOI:** 10.1093/eurpub/ckac130.101

**Published:** 2022-10-25

**Authors:** D McMenemy, MR Sweeney

**Affiliations:** School of Nursing, Psychotherapy, and Community Health, Dublin City University, Donabate, Ireland

## Abstract

**Background:**

Ireland previously had widespread voluntary fortification but there has been a major decline in the number of food staples fortified with folic acid in Irish supermarkets in the past 15 years. In this research we set out to examine the level of folic acid in food staples in supermarkets with the leading market share in the Republic of Ireland.

**Methods:**

The food labels of food staples (breads, spreads, milk, cereals, cereal bars, yoghurt/yoghurt drinks) were photographed in supermarkets with the leading market share in the Republic of Ireland (Tesco's, Dunnes, SuperValu, Lidl, Aldi, and M&S) between 2017 and 2021.The data was extracted and collated in an excel spreadsheet. The data was analysed to examine the level of folic acid in each product. We compared the levels captured at the current times with the levels previously captured in 2017.

**Results:**

Preliminary analysis suggests that folic acid level in food staples in Ireland continues to decline. Folic acid was not found in any breads (except a number of gluten free breads), milks, spreads but was found in several cereals marketed mainly at children.

**Conclusions:**

This study reports on the declining levels of folic acid in the food chain in Ireland. The number of food staples fortified with folic acid continues to decline demonstrating that voluntary fortification in Ireland is no longer an effective measure for passively augmenting the folic acid levels of consumers. This is of concern due to the incidence of neural tube defects in Ireland largely preventable by folic acid.

**Key messages:**

- This study reports on the declining levels of folic acid in the food chain in Ireland.
- The number of food staples fortified with folic acid continues to decline.

